# Enhanced polarization and abnormal flexural deformation in bent freestanding perovskite oxides

**DOI:** 10.1038/s41467-022-32519-2

**Published:** 2022-08-31

**Authors:** Songhua Cai, Yingzhuo Lun, Dianxiang Ji, Peng Lv, Lu Han, Changqing Guo, Yipeng Zang, Si Gao, Yifan Wei, Min Gu, Chunchen Zhang, Zhengbin Gu, Xueyun Wang, Christopher Addiego, Daining Fang, Yuefeng Nie, Jiawang Hong, Peng Wang, Xiaoqing Pan

**Affiliations:** 1 Department of Applied Physics, The Hong Kong Polytechnic University, Hung Hom, Kowloon 999077 Hong Kong; 2 School of Aerospace Engineering, Beijing Institute of Technology, Beijing, 100081 China; 3 National Laboratory of Solid State Microstructures, Jiangsu Key Laboratory of Artificial Functional Materials, College of Engineering and Applied Sciences and Collaborative Innovation Center of Advanced Microstructures, Nanjing University, Nanjing, 210093 China; 4 Department of Physics and Astronomy, University of California, Irvine, CA 92697 USA; 5 Institute of Advanced Structure Technology, Beijing Institute of Technology, Beijing, 100081 China; 6 State Key Laboratory for Turbulence and Complex Systems & Center for Applied Physics and Technology, College of Engineering, Peking University, Beijing, 100871 China; 7 Department of Physics, University of Warwick, Coventry, CV4 7AL UK; 8 Department of Materials Science and Engineering, University of California, Irvine, CA 92697 USA; 9 Irvine Materials Research Institute, University of California, Irvine, CA 92697 USA

**Keywords:** Ferroelectrics and multiferroics, Structural properties

## Abstract

Recent realizations of ultrathin freestanding perovskite oxides offer a unique platform to probe novel properties in two-dimensional oxides. Here, we observe a giant flexoelectric response in freestanding BiFeO_3_ and SrTiO_3_ in their bent state arising from strain gradients up to 3.5 × 10^7^ m^−1^, suggesting a promising approach for realizing ultra-large polarizations. Additionally, a substantial change in membrane thickness is discovered in bent freestanding BiFeO_3_, which implies an unusual bending-expansion/shrinkage effect in the ferroelectric membrane that has never been seen before in crystalline materials. Our theoretical model reveals that this unprecedented flexural deformation within the membrane is attributable to a flexoelectricity–piezoelectricity interplay. The finding unveils intriguing nanoscale electromechanical properties and provides guidance for their practical applications in flexible nanoelectromechanical systems.

## Abstract (web summary)

Freestanding perovskite oxides pave the way to novel flexural properties. Here, the authors observe the nanoscale strain gradient induced flexoelectric polarization with an unusual flexo-expansion/shrinkage effect in freestanding ferroelectric oxide.

## Introduction

Electromechanical properties of functional materials play a significant role in electronic devices^1,2^. For perovskite oxides with unique electromechanical functionalities, strain engineering can stimulate significant electronic phenomena^3–5^, such as inducing polarization in the nonpolar material SrTiO_3_ and attaining a record-high polarization value in PbTiO_3_^6,7^. In contrast to a homogenous strain, a strain gradient is inversely proportional to the spatial scale and can increase by seven orders of magnitude when the system shrinks from macroscale (~1/m) to nanoscale (10^7^/m)^8^. Therefore, flexoelectricity, the coupling between polarization and strain gradient, becomes a significant and even dominant effect at this nanoscale, and hence, potentially induces novel physical phenomena that have attracted much attention^9–11^. However, the simultaneous implementation of strain and strain-gradient engineering is traditionally subject to the specifications of substrates or epitaxial conditions, which largely restrict the tunability and scalability of strains and strain gradients. The recently developed sacrificial buffer-layer technique using water-soluble Sr_3_Al_2_O_6_ (SAO) provides a reliable method to synthesize high-quality freestanding thin perovskite oxides^12^. The structural stability of these perovskite oxides such as BiFeO_3_ (BFO) and SrTiO_3_ (STO) has been demonstrated in previous work^13^. Benefitting from the excellent flexibility of these oxides^14^, nanoscale mechanical bending offers a new approach in strain and strain-gradient engineering. Given that the freestanding oxides are only a few nanometers thick, they are able to generate a huge strain gradient during bending, which may induce an enhanced polarization. In addition, these unconventional low-dimensional systems may stimulate other novel physical and mechanical responses via electromechanical coupling effects (i.e., piezoelectricity and flexoelectricity)^8^, similar to their bulk counterparts, which are known to exhibit a multitude of physical properties^15^.

In addition to the novel strain-induced electrical properties of perovskite oxides^16,17^, recent studies have found some interesting mechanical properties arising from the interplay between piezoelectricity and flexoelectricity^18–20^ which implies that mechanical responses can be modulated via strain gradients. With this interplay in freestanding ultrathin films (several-unit-cell thickness), more interesting mechanical phenomena are expected in the presence of a huge strain gradient (~10^7^ m^−1^) at small scales.

In this work, we report remarkable polarization enhancements in high-quality flexible freestanding perovskite oxides of polar BFO and nonpolar STO subject to ultra-high strain gradients up to 10^7^ m^−1^ or more, revealing that the flexoelectricity plays a dominant role in determining the polarization at nanoscale. More interestingly, in addition to this enhanced polarization in BFO membranes, our results uncover unusual mechanical properties featuring a bending-expansion/shrinkage effect. This is different from the elasticity theory, in which the thickness stays constant in bent single-phase membranes. Furthermore, our analysis reveals the irregular mechanical phenomena to be driven by an interplay between flexoelectricity and piezoelectricity. Our results expose novel physical properties in bent freestanding perovskite oxides. Moreover, a new area of nanoscience is opening up, allowing the tuning of electromechanical behaviors via giant strain gradients at the atomic scale that is crucial in the related research and potential applications of nano-electromechanical systems.

## Results

### Ultra-high strain gradient at nanoscale

**Fig. 1:**
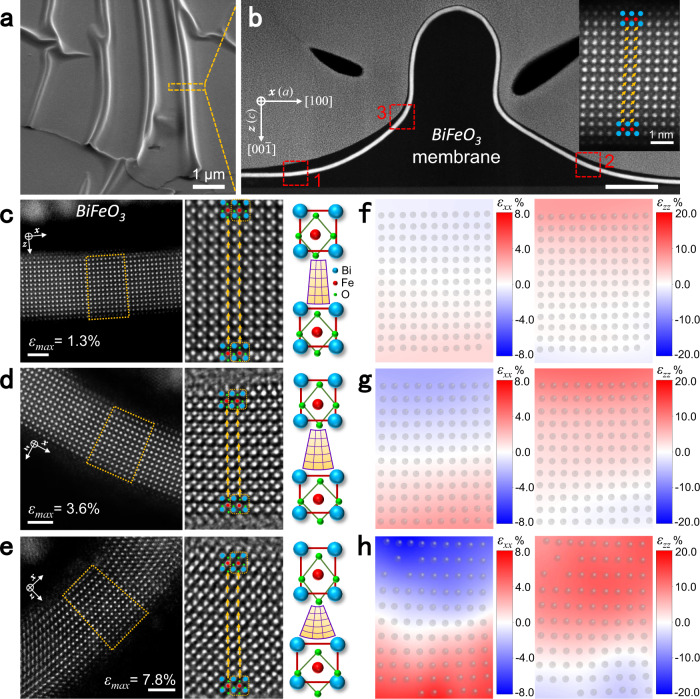
Bending behavior of BFO membranes. **a** Scanning electron microscope (SEM) image of wrinkled freestanding BFO. Scale bar: 1 μm. **b** Cross-sectional STEM-HAADF image of a single wrinkle in (**a**). Scale bar: 100 nm. Inset indicates the unstrained state taken from region 1 (marked by red square), where the spontaneous polarization has an upward out-of-plane component (yellow arrow). **c**–**e** STEM-HAADF images (left) taken from the bent regions 1, 2, 3 in (**b**), respectively. The strain gradients *ε*_*xx,z*_ are 5.2 × 10^6^ m^−1^, 2.1 × 10^7 ^m^−1^, and 3.5 × 10^7 ^m^−1^, respectively. Middle STEM-iDPC images taken from yellow trapezoid regions show the position of Bi, Fe, and O atomic columns. The variation of lattice and oxygen octahedral from the internal (IS) to external surfaces (ES) can be conjected as shown in the right schematics. Scale bar: 2 nm. **f**–**h** Corresponding mapping results of in-plane and out-of-plane strain distributions in bent regions marked by yellow trapezoids in (**c**–**e**), respectively.

**Fig. 2:**
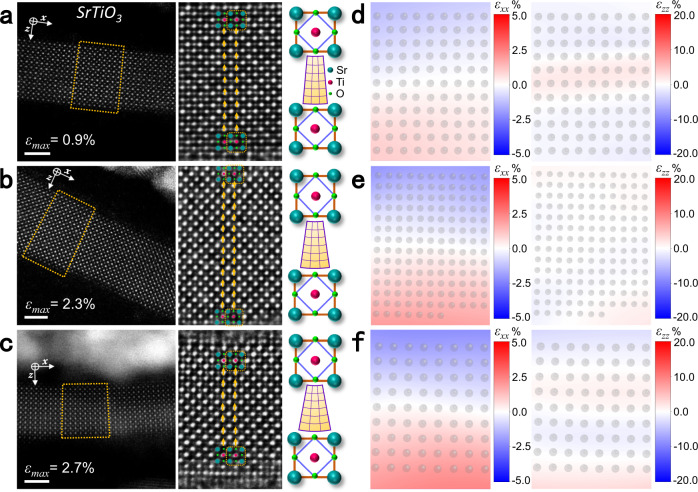
Bending behavior of STO membranes. **a**–**c** STEM-HAADF images (left) of bent STO taken from the different bent regions with a strain gradient *ε*_*xx,z*_ of 4.2 × 10^6 ^m^−1^, 9.7 × 10^6 ^m^−1^, and 1.5 × 10^7 ^m^−1^, respectively. Middle STEM-iDPC images taken from yellow trapezoid regions show the position of Sr, Ti, and O atomic columns. The variation of lattice and oxygen octahedral from the internal (IS) to external surfaces (ES) can be conjected as shown in the right schematics. Scale bar: 2 nm. **d**–**f** Corresponding mapping results of in-plane and out-of-plane strain distributions in bent regions marked by yellow trapezoids in (**a**–**c**), respectively.

Freestanding ultrathin BFO and STO (~5-nm thickness) with corrugations were fabricated (Fig. 1a) and further prepared as cross-sectional transmission electron microscopy (TEM) samples (details in “Methods” and Supplementary Figs. 1–4). Atomic-resolution scanning TEM high-angle annular dark-field (STEM-HAADF) images of BFO (Fig. 1c–e) and STO (Fig. 2a–c) were acquired from different bent regions. The lattice structures have maintained their integrity and continuity without any obvious rupture even under a strain up to 7.8% and 2.7% at surfaces in the BFO and STO membranes, indicating the high flexibility of freestanding thin perovskite oxides (for STO, higher strain has also been observed as shown in Supplementary Fig. 5). We observed a huge strain gradient *ε*_*xx,z*_ arising from a considerable change in the in-plane strain corresponding to variations in lattice spacings *a* (Figs. 1f–h, 2d–f) across the membrane in the nanometer range. Here, we call the surface of a bent membrane facing toward the center of curvature (Supplementary Fig. 6) the internal surface (IS), which is subject to the compressive in-plane strain (negative values), and the opposing surface the external surface (ES), which is subject to the tensile in-plane strain (positive values). The maximum strain gradient of the BFO (Fig. 1e) and STO (Fig. 2c) membranes are up to ~3.5 × 10^7 ^m^−1^ and ~1.5 × 10^7 ^m^−1^, respectively, which are nearly one order of magnitude larger than that generated in the epitaxial films (10^5^–10^6 ^m^−1^)^9,10,21^. Although similar strain gradients were observed just under the tip of an atomic force microscope (10^6^–2 × 10^7 ^m^−1^)^22,23^, the uniformity of this huge strain gradient across the entire thickness range is a unique property of the bent freestanding membranes, which offers better tunability in flexoelectric applications.

### Polarization in bent perovskite oxide membranes

The polarization evolution (marked by yellow arrows in Figs. 1c–e and 2a–c according to B-site cation displacement) in BFO and STO membranes are associated with the lattice distortions. To understand the relationship between the enhanced strain gradient and the corresponding polarization at the nanoscale, quantitative probing of the polarization at single unit cell level is essential but remains challenging. Here, we first used a widely accepted empirical method assuming that the polarization magnitude in ABO_3_ perovskites is proportional to the off-centering displacement (δ*z*) of the B site cation with respect to the center of four surrounding A site cations (Supplementary Fig. 7b)^7,24–26^. This simple yet direct semiquantitative method exhibits a widely acceptable accuracy even for large polarization up to 236 μC/cm^2^ in previous study^7^. Next, a recently developed integrated differential phase contrast (iDPC) imaging method was applied to reveal the position of cations and oxygen octahedrons (middle insets in Figs. 1c–e, 2a–c)^27^. As all the cation-anion bond lengths are measurable in STEM-iDPC images, the relative atomic displacements δ_*i*_ of all ions can be determined. Therefore, the polarization magnitude can be calculated through the following relationship: *P* = (Σδ_*i*_*Z*_*i*_)/*V*, where *V* is the volume of a single unit cell (in our case we use *a*^2^*c*), δ_*i*_ is the displacement of ion *i* and *Z*_*i*_ is the Born effective charge (BEC) of ion *i*^27^ (Supplementary Fig. 7, details in “Methods”). The polarization values acquired by these two approaches exhibit only slight differences, no more than 15% (Supplementary Fig. 11). In addition, we performed the first-principles calculations to investigate the polarization evolution of bulk BFO with corresponding δ*z*. The result shows an almost linear relationship between the polarization and δ*z* of the B site cation, even in the large displacement value of 1.1 Å (details in “Methods” and Supplementary Fig. 8), suggesting the rationality of polarization calculation.

In unstrained ferroelectric BFO, we found the off-centering displacement of Fe cations (δ*z*-Fe) to be oriented diagonally and the magnitude of the out-of-plane displacement to be nearly uniform across the membrane (inset in Fig. 1b and Supplementary Figs. 3, 7). However, after bending, the off-centering displacement is oriented mostly along the thickness direction and decrease from the ES (tensile in-plane strain) to the IS (compressive in-plane strain); see Fig. 1c–h and Supplementary Fig. 9. Interestingly, for the bent freestanding STO, the Ti cations also undergo an off-centering displacement (δ*z*-Ti) oriented along the thickness direction, as shown in Fig. 2a–c. Unlike the BFO cases, the displacement δ*z*-Ti in STO remains almost constant across the membrane thickness, and increases with increasing strain gradient (Supplementary Fig. 10).

**Fig. 3:**
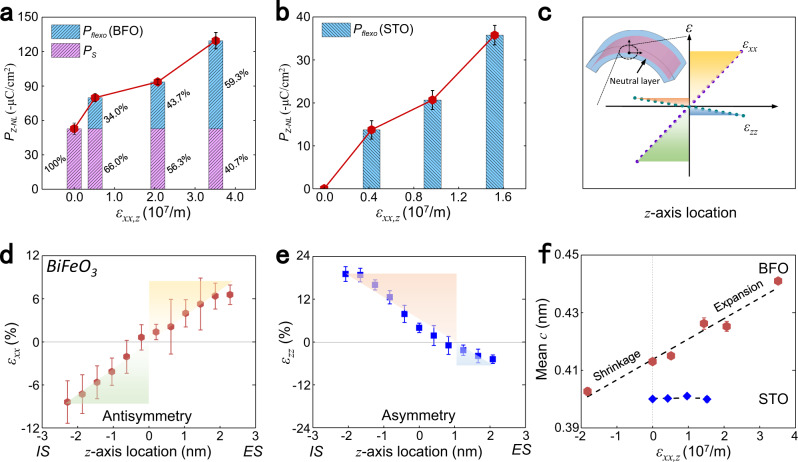
Mechanical behavior of bent BFO and STO membranes. **a**, **b** The out-of-plane polarizations at the neutral layer and their corresponding strain gradients from the bent BFO and STO membranes, respectively. Error bar represents the standard deviation of measured unit-cells. **c** Schematic of the theoretical antisymmetric in-plane and out-of-plane strain distributions in the bent membrane. **d** Antisymmetric in-plane and (**e**) asymmetric out-of-plane strain distributions in the bent BFO with a strain gradient of 3.5 × 10^7 ^m^−1^ along the thickness direction. Error bar represents the standard deviation of measured unit-cells. **f** Mean lattice spacing *c* as a function of the strain gradient in the bent BFO and STO. Error bar represents the standard deviation of measurement in each case.

With the off-centering displacement of B-site cations and cation-anion bond lengths measured from the STEM-iDPC images, we obtained the polarization distributions along the thickness direction in bent freestanding BFO and STO membranes using two approaches as demonstrated before (Supplementary Fig. 11). Several features were noted: (1) the larger strain and strain gradient will subsequently produce larger polarizations in both BFO and STO. (2) The bent STO has a near uniform distribution in polarization across the membrane (Fig. 2a–c and Supplementary Fig. 11), while the polarization increases from IS to ES in the bent BFO, possibly arising from its piezoelectricity. This is confirmed from our phase-field simulations (Supplementary Fig. 12), and (3) the maximum polarization occurs in a bent membrane that possesses the largest strain gradient. For example, for the bent BFO under a strain gradient of 3.5 × 10^7 ^m^−1^, the maximum polarization reached was ~146.6 ± 5.3 μC/cm^2^ in magnitude at the external layer (Supplementary Fig. 13), which is 2.8 times stronger than the spontaneous polarization of 52.7 ± 7.5 μC/cm^2^ measured from the unstrained BFO membrane (Supplementary Fig. 7). This magnitude is larger than that in a tetragonal-like BFO film (130 μC/cm^2^)^28^. For the bent STO with a strain gradient up to 1.5 × 10^7 ^m^−1^, its polarization magnitude reaches ~35.3 ± 8.5 μC/cm^2^ cross the membrane thickness (Fig. 3b and Supplementary Fig. 11).

### Large flexoelectricity induced by strain gradient

The enhanced polarization observed in these bent freestanding perovskite oxides should be attributed to the strain-induced piezoelectricity (polar BFO) and strain gradient-induced flexoelectricity (both polar BFO and nonpolar STO); in particular, the latter provides a major contribution in such extreme strain-gradient conditions. The total out-of-plane polarization *P*_*z*_ originates from out-of-plane spontaneous ${P}_{s\perp }$, piezoelectric and flexoelectric polarizations; that is, ${P}_{z}={P}_{s\perp }+{\widetilde{e}}_{{zxx}}{\varepsilon }_{{xx}}+{\widetilde{\mu }}_{{zxxz}}{\varepsilon }_{{xx},z}$, where ${\widetilde{e}}_{{zx}x}$ and $\,{\widetilde{\mu }}_{{zxxz}}$ are the coefficients of the effective transverse piezoelectricity and flexoelectricity, and *ε*_*xx*_ and *ε*_*xx,z*_ are the in-plane strain and its gradient along the thickness direction. In the calculations of polarizations and strain gradients throughout this work, we define the [00$\bar{1}$] crystal direction of the membranes as the positive direction (Figs. 1 and 2); further details are given in Supplementary Fig. 6.

For simplification, the neutral layer where the in-plane strain is near zero (*ε*_*xx*_ = 0) is chosen in the analysis of the flexoelectric polarization to strain gradient. The polarization at the neutral layer *P*_*z*-NL_ is deduced as ${P}_{z}={P}_{s\perp }+{\widetilde{\mu }}_{{zxxz}}{\varepsilon }_{{xx},z}$. The neutral layer polarization *P*_*z*-NL_ of BFO increases almost linearly with the enhancement of strain gradient, as shown in Fig. 3a. Note that the flexoelectric contribution to polarization is more pronounced as the strain gradient increases. In particular, when the strain gradient reaches 3.5 × 10^7^ m^−1^, flexoelectricity offers more than 50% enhancement of the polarization at the neutral layer. This pronounced flexoelectric polarization thus contributes ~52.4% of the maximum polarization at the external layer of bent BFO (Supplementary Fig. 13). The effective transverse flexoelectric coefficient ${\widetilde{\mu }}_{zx{xz}}$ was calculated as −19.4 ± 3.5 nC/m with the slope of the fitted line of Fig. 3a (the top surface of BFO membrane used here is terminated on a Bi-O layer while bottom surface is terminated on a Fe-O layer). The negative sign of the coefficient indicates that the direction of the flexoelectric polarization is opposite to the strain gradient and always points toward the center of curvature (see Supplementary Fig. 6). It is worth noting that this strain gradient-driven *P*_*z*_ enhancement (flexoelectric contribution) may have several physical origins in BFO, including the possible polarization rotation. As shown in Fig. 1c, with the increase of *P*_*z*_ from IS to ES, a decreasing trend appears in the in-plane polarization component, which implies a potential in-plane to out-of-plane rotation of spontaneous polarization. For bent nonpolar STO, the polarization stems entirely from the flexoelectricity, which also exhibits the similar strain gradient-dependent trend as in bent BFO (Fig. 3b). The flexoelectric coefficient ${\widetilde{\mu }}_{zx{xz}}$ of STO was calculated as −22.2 ± 2.4 nC/m (both the top and bottom surfaces of STO membrane used here are Sr-O terminated; see Supplementary Fig. 14)^29^. The magnitude of these coefficients matches well with those of other ferroelectric materials predicted from the first-principles methods^11^. In summary, since a bent perovskite oxide membrane is capable of accommodating huge strain gradients, the corresponding flexoelectric polarization can be large enough to dominate the localized polarization.

### Flexo-expansion and shrinkage effects in ferroelectric membranes

The huge strain gradient in bent freestanding perovskites not only induces an enhanced polarization, but also drives an unusual “bending-expansion” behavior. Classical elastic bending theory assumes that the in-plane and out-of-plane strains have antisymmetric distributions across the bent membrane (Fig. 3c). Indeed, the strain distributions of *ε*_*xx*_ and *ε*_*zz*_ in bent STO (Fig. 2d–f and Supplementary Fig. 16), as well as the in-plane strain *ε*_*xx*_ in bent BFO (Fig. 1f–h and Supplementary Fig. 15a–c), roughly agree with this theory despite the thickness of these membranes being only several nanometers. However, the out-of-plane strain *ε*_*zz*_ in bent BFO was found to be significantly asymmetric (Fig. 1f–h and Supplementary Fig. 15d–f). The tensile strain region (triangle area in yellow color) becomes much larger than the compressive strain region (triangle area in bule color) as the strain gradient increases (Supplementary Fig. 15d–f). The tensile strain region almost dominates across the membrane when the strain gradient is up to 3.5 × 10^7^ m^−1^ as shown in Fig. 3e. Consequently, this asymmetric out-of-plane strain distribution induces an abnormal change in membrane thickness under bending (herein, referred to as flexoexpansion or flexoshrinkage). The mean lattice spacing *c* in bent BFO indeed increases (flexoexpansion) under a positive strain gradient, but remains constant in bent STO duo to its symmetric strain distributions (Fig. 3f). The change in BFO membrane thickness is proportional to the strain gradient, leading to the overall thickness of BFO increasing by 6.8% as the strain gradient reaches 3.5 × 10^7 ^m^−1^.

To explain the flexoexpansion in bent BFO, we developed an electromechanical model (details are given in “Methods”). The expression for the thickness of the bent membrane *h* is as follows:

1 $$h=(A{\varepsilon }_{xx,z}+1){h}_{0},$$

2 $$A=\frac{{d}_{zxx}{F}_{zzzz}-{d}_{zzz}{F}_{zxxz}}{{s}_{xxxx}{k}_{zz}-{d}_{zxx}^{2}},$$

where *h*_0_ denotes the thickness of the flat membrane, *s*_*ijkl*_, *d*_*ijk*_, *F*_*ijkl*_, and *k*_*ij*_ denote the elastic compliance, piezoelectric, flexoelectric, and dielectric tensors, respectively. From Eqs. (1) and (2), the thickness depends linearly on the strain gradient. These abnormal trends exhibit a dependence on coefficient *A*, which is nonzero only when the material manifests piezoelectric and flexoelectric effects simultaneously. Our model indicates that the interplay between flexoelectricity and piezoelectricity provides a biased electromechanical out-of-plane strain, which is explained in detail in “Methods”. This explains why BFO shows a flexoexpansion effect, but STO does not due to its lack of a piezoelectric effect.

**Fig. 4:**
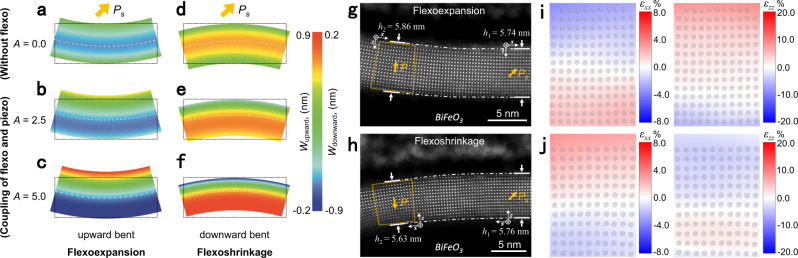
Flexoexpansion and flexoshrinkage effects in piezoelectric membranes. **a**–**f** Simulated bending deformation of ferroelectric membranes (5 nm thickness) with different bending expansion coefficients *A* = 0.0 (**a**, **d**), 2.5 (**b**, **e**), and 5.0 (**c**, **f**) when bent upward (**a**–**c**) and downward (**d**–**f**), given that the out-of-plane spontaneous polarization (yellow arrow) points upward. **g**, **h** The flexoexpansion and flexoshrinkage effects observed in upward (**g**) and downward (**h**) bent BFO, respectively. **i**, Strain mapping of trapezoid region in (**g**) shows the symmetric in-plane strain distribution and asymmetric out-of-plane strain distribution along the thickness direction. **j** Strain mapping of trapezoid region in (**h**) shows the symmetric in-plane strain distribution and asymmetric out-of-plane strain distribution opposite with the case in (**i**).

A value for the linear coefficient *A* of 1.7 ± 0.2 nm for BFO was obtained by fitting Eq. (1) to the data (Fig. 3f). This value of *A* = 1.7 nm indicates that the thickness varies by 1.7% per 1 × 10^7^ m^−1^ of strain gradients and also explains why flexoexpansion has never been observed at the macroscopic scale, on which the strain gradient normally is only of order 10 m^−1^. When the membrane is bending in the [00$\bar{1}$] direction (i.e., positive *z* direction; see Supplementary Fig. 6), the sign of the strain gradient *ε*_*xx,z*_ is reversed as the strain decreases along the [00$\bar{1}$] direction. Therefore, in accordance with Eq. (1), the thickness of the membrane becomes shortened (flexoshrinkage). Both flexoexpansion and flexoshrinkage can be predicted from a theoretical perspective (Fig. 4a–f). In the experiment, we indeed, also found flexoshrinkage occurring in an oppositely bent BFO membrane (Fig. 4h, j, and Supplementary Fig. 18), while the positive bent BFO maintains flexoexpansion (Fig. 4g, i, and Supplementary Fig. 17), thereby validating our model. Interestingly, this asymmetrically distributed out-of-plane strain (Fig. 4j, and Supplementary Fig. 18c) is inversely symmetric with those for upwardly bent membranes (Fig. 3e and Supplementary Fig. 15d–f). The expansion or shrinkage across the membranes in different bending directions indicates that the polar freestanding oxides possess an asymmetric bending rigidity, which must be taken into account in future studies and applications.

## Discussion

In summary, the freestanding perovskite oxides exhibit an exceptional flexibility and capability to accommodate a giant strain gradient. The flexoelectric polarization becomes so predominant at atomic scale that strain gradient engineering offers a new path toward manipulating electrical and mechanical behaviors in these strongly correlated two-dimensional materials as future potential building blocks in multifunctional flexible electronics^30–32^ and nanomachines^2^. For example, the strong strain gradient within self-rolling heterostructures provides a powerful tool of flexoelectric polarization for designing novel energy harvesters and field-effect transistors^33,34^. Furthermore, the enhanced flexoelectric polarization opens a door for the application of intrinsic nonpolar materials in polarity-dependent electronics. We discovered in experiments that the interplay between flexoelectricity and piezoelectricity leads to an asymmetric change in bending-thickness with respect to the sign of the spontaneous polarization in low-dimensional polar material. Given that the bending rigidity is sensitive to the thickness change, the strain gradient-driven flexo-expansion or flexo-shrinkage phenomenon will lead to an asymmetric bending rigidity with respect to the sign of the spontaneous polarization. That means the bending rigidity possess two different states, “rigid” state by flexo-expansion and “soft” state by flexo-shrinkage. These two states can be reversibly switched by modulating the direction of spontaneous polarization with an electric field. Therefore, the freestanding ferroelectric oxides can serve as a type of variable-stiffness electromechanical operator, which provide a promising approach in controlling the configurations of 3D buckling self-assembled nano-structures^35,36^ and programmable shape morphing devices^37^. This unusual mechanical property is expected to make BFO and other ferroelectric membranes effective smart mechanical materials^18^ and strongly influence nano-mechanical performances regarding, for example, the vibration, fracture, and wrinkling modes that play a crucial role in applications of nano-electromechanical systems and three-dimensional self-assembled nano-structures^36,38^.

## Methods

### Epitaxial film growth and transfer

Water-soluble SAO layer was grown first on (001) STO single crystalline substrate followed by the growth of a thin film (STO or BFO) by Oxide-MBE. The SAO films were grown with an oxidant (10% O_3_ and 90% O_2_) background pressure ${p}_{{{{{{{\rm{O}}}}}}}_{2}}$ of 1 × 10^−6^ Torr and at a substrate temperature *T*_g_ of 750 °C. The STO films were grown with ${p}_{{{{{{{\rm{O}}}}}}}_{2}}$ = 1 × 10^−6^ Torr and at *T*_substrate_ = 650 °C. The SAO and STO films were grown in a layer-by-layer growth mode, for which the thickness was monitored by RHEED oscillations. The BFO films were grown with an oxidant (distilled O_3_) background pressure of 1 × 10^−5^ Torr and at *T*_substrate_ = 380 °C. Due to the volatility of bismuth, BFO films were grown in adsorption-controlled mode with a fixed Bi:Fe flux ratio of 7:1 and the thickness was controlled by shutter time of iron. The RHEED electron beam was blanked during the growth of BFO films to improve the film quality. To transfer the freestanding oxide film to a silicon substrate, the sample was adhered onto PDMS or silicone/PET and then immersed in deionized water. After dissolving SAO in water, the film/PDMS or film PET/silicone/PET was attached onto the new substrate. Finally, the freestanding film remained on the new substrate after peeling off the PDMS or silicone/PET. After a mechanical transfer procedure, some regions of freestanding films exhibit regular wrinkled structure. These wrinkle stripes have a typical width of several hundred nanometers with a height at same level.

### TEM cross-sectional sample preparation and SEM imaging

High-quality cross-sectional samples were fabricated by focused ion beam (FIB) technique using FEI Helios 600i dual-beam system. First, the regions of wrinkles along [100] or [010] direction on a transferred freestanding perovskite oxide film were located using SEM. Second, a 300 nm thick Pt protection layer was deposited on the freestanding film surface using an electron beam of 5.5 nA current at an accelerating voltage of 2 kV, and followed by a 3 μm thick deposited Pt protection layer with a gallium ion beam. Third, cross-sectional lamellas were formed by gallium ion beam etch and then transferred onto TEM grids by in situ lift-out system. The cross-sectional lamellas were further thinned by gallium ion beam at an accelerating voltage of 30 kV with 0.79 nA to 80 pA beam current. Finally, a gentle ion milling procedure using a 2 kV accelerating voltage with a beam current of several tens pico amps was employed to reduce the superficial amorphous layers induced by ion implantation damage. SEM images were acquired on FEI Helios 600i dual-beam system using an electron beam of 43 pA current at an accelerating voltage of 2 kV.

### STEM imaging methods and data processing

Atomic resolution STEM-HAADF images were obtained on a double aberration-corrected S/TEM Thermofisher Spectra 300 at 300 kV with a field emission gun. The probe convergence angle was 24.5 mrad, and the angular range of the HAADF detector was from 79.5 mrad to 200 mrad. iDPC data were also collected on the same microscope with a 24.5 mrad convergence angle using 8 segments annular detector, which exhibits a higher contrast on oxygen anions.

4D-STEM data in Supplementary Fig. 7 were collected on a double aberration-corrected S/TEM Thermofisher Titan G2 at 300 kV with a 22.5 mrad convergence angle. The diffraction patterns of the 4D-STEM datasets were recorded with a 128 × 128 pixel array detector (EMPAD) at an acquisition rate of 1000 frames per second. The scanning area of 2.6 × 2.6 nm was acquired with a scanning step size of 0.2 Å. Maximum collection semi-angle of the EMPAD detector was 67 mrad.

A center of mass (COM) signal image can be obtained directly as the center of mass motion is calculated from each diffraction pattern in the 4D-STEM dataset. A differentiated COM (dCOM) signal image is generated by calculating the divergence of the COM image^39^.

### Lattice and polarization measurements

To determine the atom positions from the STEM images, we extracted the intensity line profile of each unit cell layer and define the position with the highest intensity in a single atom region as the center of this atom. Then we use this position to calculate the space between two neighboring A-site cations and get the lattice spacing (Supplementary Figs. 9 and 10). For mapping the strain distribution, we use Gaussian Fitting to find the positions of A-site cations and automatically calculate the relative spacing of neighboring cations in the in-plane and out-of-plane directions. The difference between these spacing values and reference values on unstrained state can be calculated as corresponding strain magnitude.

For semiquantitative analysis of the out-of-plane polarization based on the STEM-iDPC images, we used the relative displacement of B site cation column to center of nearest four A site cation columns (Supplementary Fig. 7b). The procedure was based on the empirical formula *P* = *k*δ*z*, where *P* is the polarization, *k* is an empirical constant fitted from macroscopic measurement of corresponding ferroelectric materials, δ*z* is the displacement of B site cation to A site cations.

For precise determination of the out-of-plane polarization, a recently developed method, in which the calculations of the displacement include A site cation, B site cation, and oxygen anion positions, provides more accurate polarization measurements. However, this technique relies on atomically resolved STEM-iDPC data. Here, atomically resolved STEM-iDPC images were utilized to reveal the position of cations, oxygen octahedrons, and the length of cation-anion bonds. Bond length *d*_*i*_ is the distance between two adjacent atoms, here we only consider the distance along out-of-plane direction. The relative atomic displacement δ_*i*_ was calculated through the formula (*d*_*i+*1_ − *d*_*i*_)/2 (*d*_*i*_ and *d*_*i+*1_ are the adjacent bond lengths). Then the polarization value was calculated through the following precise relationship: *P* = (Σδ_*i*_*Z*_*i*_)/*V*, where *V* is the volume of a single unit cell (in our case we use *a*^2^*c*), δ_*i*_ is the displacement of atom *i* from its centrosymmetric position, *Z*_*i*_ is the BEC of cation *i*^27^. Here we used the numerical value of BEC calculated by ab initio theory as 3.49 for Fe, 4.37 for Bi^40^, 7.12 for Ti, and 2.54 for Sr^41^.

### Calculation of flexoelectric coefficient

In this study, the total out-of-plane polarization *P*_*z*_ measured in bent BFO membrane can be simplified as the sum of the out-of-plane spontaneous polarization ${P}_{s\perp }$, the piezoelectric polarization caused by strain, and the flexoelectric polarization driven by strain gradient:

3 $${P}_{z} 	=\, {P}_{s\perp }+{e}_{zxx}{\varepsilon }_{xx}+{e}_{zzz}{\varepsilon }_{zz}+{\mu }_{zxxz}{\varepsilon }_{xx,z}+{\mu }_{zzzz}{\varepsilon }_{zz,z}\\ 	 \,={P}_{s\perp }+{\tilde{e}}_{zxx}{\varepsilon }_{xx}+{\tilde{\mu }}_{zxxz}{\varepsilon }_{xx,z}.$$

The subscript “⊥” represents the polarization component along the *z*-axis direction. We used the effective transverse electromechanical coefficients ${\tilde{e}}_{zxx}={e}_{zxx}-{v}_{zx}{e}_{zzz}$ and ${\tilde{\mu }}_{zxxz}={\mu }_{zxxz}-{v}_{zx}{\mu }_{zzzz}$ (where *v*_*zx*_ is Poisson’s ratio) to characterize the piezoelectric and flexoelectric effect, respectively. *ε*_*xx*_ and *ε*_*xx,z*_ are the in-plane strain and its gradient along the *z*-axis direction, respectively.

The in-plane strain *ε*_*xx*_ is approximated as being anti-symmetrically distributed along the *z*-axis direction in the bent membrane (Fig. 3d and Supplementary Fig. 14), so the neutral layer (NL) of the bent membrane in which there is no in-plane strain (*ε*_*xx*_ = 0) has no piezoelectric polarization. The Eq. (3) for the neutral layer can be simplified as

4 $${P}_{z-NL}={P}_{s\perp }+{\tilde{\mu }}_{zxxz}{\varepsilon }_{xx,z}.$$

Thus, the flexoelectric coefficient ${\widetilde{\mu }}_{{zxxz}}$ can be determined by fitting the polarization at the neutral layer and the strain gradient. As shown from experiment observation (Supplementary Figs. 14 and 15), the strain is almost linearly distributed along thickness direction, suggesting the strain gradient is nearly constant.

### Theoretical analysis for irregular mechanical property

The flexoelectric theoretical framework for dielectrics is applied to investigate the mechanism underlying the bending-expansion or -shrinking behavior in BFO membrane. Taking the piezoelectric and flexoelectric effect into account, the expression for the Gibbs free energy density of dielectrics can be written as^42,43^

5 $$G=\frac{1}{2}{k}_{ij}{E}_{i}{E}_{j}+\frac{1}{2}{s}_{ijkl}{\sigma }_{ij}{\sigma }_{kl}+{d}_{kij}{\sigma }_{ij}{E}_{k}+{F}_{kijl}\left({E}_{k}\frac{\partial {\sigma }_{ij}}{\partial {x}_{l}}-{\sigma }_{ij}\frac{\partial {E}_{k}}{\partial {x}_{l}}\right)-{D}_{i}{E}_{i}-{\sigma }_{ij}{\varepsilon }_{ij},$$

where *E*_*i*_ and *D*_*i*_ are the electric field and the electric displacement tensors, respectively; *σ*_*ij*_ and *ε*_*ij*_ are the stress and the strain tensors; *k*_*ij*_, *s*_*ijkl*_, *d*_*ijk*_, and *F*_*ijkl*_ are the second-rank dielectric permittivity tensor, the fourth-rank elastic compliance tensor, the third-rank piezoelectric coupling tensor, and the fourth-rank flexoelectric coupling tensor, respectively.

The electromechanical constitutive equations can be obtained by minimizing the Gibbs free energy:

6 $${D}_{k}={k}_{kl}{E}_{l}+{d}_{kij}{\sigma }_{ij}+{F}_{kijl}\frac{\partial {\sigma }_{ij}}{\partial {x}_{l}},$$

7 $${\varepsilon }_{ij}={s}_{ijkl}{\sigma }_{kl}+{d}_{kij}{E}_{k}-{F}_{kijl}\frac{\partial {E}_{k}}{\partial {x}_{l}}.$$

The total polarization of each lattice almost points to the *z*-axis direction. According to Gauss’s law, the electric displacement along the *z*-axis direction should satisfy the following equation:

8 $${D}_{z,z}=0.$$

Substituting Eq. (6) into Eq. (8), the electric field and its gradient along the *z*-axis direction induced by bending can be obtained:

9 $${E}_{z}=-\frac{{d}_{zxx}}{{k}_{zz}}{\sigma }_{xx}-\frac{{F}_{zxxz}}{{k}_{zz}}\frac{\partial {\sigma }_{xx}}{\partial {x}_{z}},$$

10 $$\frac{\partial {E}_{z}}{\partial {x}_{z}}=-\frac{{d}_{zxx}}{{k}_{zz}}\frac{\partial {\sigma }_{xx}}{\partial {x}_{z}}-\frac{{F}_{zxxz}}{{k}_{zz}}\frac{{\partial }^{2}{\sigma }_{xx}}{\partial {x}_{z}^{2}}.$$

From Eqs. (9)–(10), the flexoelectric effect produces a bias electric field, while the piezoelectric effect induces an electric field gradient across the BFO membranes. Substituting Eqs. (9)–(10) into Eq. (7), the in-plane strain and out-of-plane strain are derived as:

11 $${\varepsilon }_{xx}=\left({s}_{xxxx}-\frac{{d}_{zxx}^{2}}{{k}_{zz}}\right){\sigma }_{xx}+\frac{{F}_{zxxz}^{2}}{{k}_{zz}}\frac{{\partial }^{2}{\sigma }_{xx}}{\partial {x}_{z}^{2}},$$

12 $${\varepsilon }_{zz}=\left({s}_{zzxx}-\frac{{d}_{zzz}{d}_{zxx}}{{k}_{zz}}\right){\sigma }_{xx}+\frac{{d}_{zxx}{F}_{zzzz}-{d}_{zzz}{F}_{zxxz}}{{k}_{zz}}\frac{\partial {\sigma }_{xx}}{\partial {x}_{z}}+\frac{{F}_{zzzz}{F}_{zxxz}}{{k}_{zz}}\frac{{\partial }^{2}{\sigma }_{xx}}{\partial {x}_{z}^{2}}.$$

According to the experiments’ results, the in-plane strain *ε*_*ij*_ is approximated as anti-symmetrically distributed along the *z*-axis direction in bent BFO membrane (Fig. 3d and Supplementary Figs. 14 and 15), implying that the second term at the right side of Eq. (11) can be ignored. Therefore, the in-plane stress *σ*_*ij*_ is considered as anti-symmetrically and linearly distributing along the *z*-axis direction for the sake of satisfying the stress equilibrium. To simplify the derivation, the second term in Eq. (11) and the third term in Eq. (12) are neglected in following derivation.

Equation (12) indicates that the coupling of flexoelectricity and piezoelectricity provides a bias electromechanical out-of-plane strain, which essentially consists of two parts: the nanoscale enhanced flexoelectric effect triggers a large out-of-plane electric field, leading to an extra out-of-plane strain by the inverse piezoelectric effect; also the piezoelectric effect results in a large electric field gradient, which generates another extra out-of-plane strain by the inverse flexoelectric effect.

The relationship between the thickness and strain gradient is obtained by combining Eqs. (11) and (12):

13 $$h=(A{\varepsilon }_{xx,z}+1){h}_{0},$$

14 $$A=\frac{{d}_{zxx}{F}_{zzzz}-{d}_{zzz}{F}_{zxxz}}{{s}_{xxxx}{k}_{zz}-{d}_{zxx}^{2}},$$

where *h*_0_ denotes the thickness of the flat membrane. Based on the experimental results (Fig. 3f), the coupling coefficient *A* of BFO membrane are calculated as 1.7 ± 0.2 nm, without using all the tensor components in Eq. (14), the measurement of which are challenging to obtain at the nanoscale.

### The phase-field computational methods

Phase-field simulations were performed to investigate the polarization state in the bent BFO membranes. The temporal evolution of the polarization field is described by the time-dependent Ginzburg–Landau (TDGL) equations:

15 $$\frac{\partial P({{{{{\boldsymbol{r}}}}}},t)}{\partial t}=-L\frac{{\delta }F}{{\delta }P({{{{{\boldsymbol{r}}}}}},t)},i=1,2,3,$$

where *Pi*(*r*, *t*) is polarization, *r* is the spatial coordinate, *t* is the evolution time, *L* is the kinetic coefficient, and *F* is the total free energy that includes the contributions from the bulk energy, the Landau energy, the gradient energy and the flexoelectric field energy^44^:

16 $$F=\iiint ({f}_{{{{{{\rm{bulk}}}}}}}+{f}_{{{{{{\rm{Land}}}}}}}+{f}_{{{{{{\rm{grad}}}}}}}+{f}_{{{{{{\rm{flexo}}}}}}})dV.$$

The bulk energy density *f*_bulk_ is described as follows,

17 $${f}_{{{{{{\rm{bulk}}}}}}}=\frac{1}{2}{c}_{ijkl}({\varepsilon }_{ij}-{\varepsilon }_{ij}^{0})({\varepsilon }_{kl}-{\varepsilon }_{kl}^{0})-{e}_{ijk}^{T}{E}_{k}({\varepsilon }_{ij}-{\varepsilon }_{ij}^{0})-\frac{1}{2}{\varepsilon }_{0}{\kappa }_{ij}{E}_{i}{E}_{j},$$

where *c*_*ijkl*_ and ${e}_{ijk}^{T}$ are the elastic stiffness tensor and piezoelectric stiffness tensor, respectively. *ε*_*ij*_ and ${\varepsilon }_{ij}^{0}$ are the total local strain and eigenstrain, respectively, *E*_*i*_ is the electric field component, *ε*_0_ is the vacuum permittivity, and *κ*_*ij*_ is the background dielectric constant.

The Landau energy density *f*_Land_ is expressed as:

18 $${f}_{{{{{{\rm{Land}}}}}}}={\alpha }_{ij}{P}_{i}{P}_{j}+{\alpha }_{ijkl}{P}_{i}{P}_{j}{P}_{k}{P}_{l},$$

where *α*_*ij*_ is the Landau energy coefficients. The gradient energy density *f*_grad_ is given by,

19 $${f}_{{{{{{\rm{grad}}}}}}}=\frac{1}{2}{G}_{ijkl}{P}_{i{,}j}{P}_{k{,}l}$$

where *G*_*ijkl*_ is the gradient energy coefficient. The flexoelectric field energy density *f*_flexo_ is given by,

20 $${f}_{{{{{{\rm{flexo}}}}}}}=-{E}_{k}^{f}{P}_{k}$$

where ${E}_{k}^{f}=-\frac{\delta {f}_{{{{{{\rm{flexo}}}}}}}}{\delta {P}_{k}}={f}_{ijkl}\frac{\partial {\varepsilon }_{ij}}{\partial {x}_{l}}$ and *f*_*ijkl*_ is the flexoelectric coefficients^45^.

In the simulations, the BFO membrane model was discretized at grid size 120 Δ*x* × 10Δ*x*, where Δ*x* was set to 0.5 nm. The total thickness is about 5.0 nm, which is consistent with the thickness of the specimen measured in the experiments. The open circuit condition was applied along the boundaries *z* direction of the thin membrane, and the temperature was set to be 298 K. The Values of the parameters in the simulations are also listed in Supplementary Table 1^46,47^.

### The first-principles calculations of BFO polarization

The corresponding calculations were carried out by the generalized gradient approximation (GGA) method of Perdewe–Burke–Ernzerhof (PBE)^48^ based on density functional theory (DFT) implemented in the Vienna ab initio Simulation Package (VASP)^49,50^. The cutoff energy for the plane wave basis set was tested and taken as 500 eV. Both lattice constants and atomic positions were relaxed until the forces on atoms were <0.005 eV/Å, and the total energy change was <10^−5^ eV. The polarization evolution of bulk BFO corresponding Fe-displacement (Supplementary Fig. 8) was calculated by the Berry phase method^51,52^.

## Supplementary information

Supplementary Information

## Data Availability

The authors declare that data supporting the findings of this study are available within the paper and its Supplementary Information files. The experiment raw data of this study are available from the corresponding author upon reasonable request.
