# Supplementary material for: Enhanced polarization and abnormal flexural deformation in bent freestanding perovskite oxides
Source: Nat Commun. 2022 Aug 31;13:5116. doi: 10.1038/s41467-022-32519-2 (PMC9433432; doi:10.1038/s41467-022-32519-2)
Supplement: Supplementary file 1 — Supplementary Information [file 41467_2022_32519_MOESM1_ESM.pdf]

Supplementary Information

Enhanced Polarization and abnormal flexural deformation in bent freestanding perovskite oxides

Songhua Cai[1,#,*], Yingzhuo Lun[2,#], Dianxiang Ji[1,#], Peng Lv[2], Lu Han[3], Changqing Guo[2], Yipeng Zang[3], Si Gao[3], Yifan Wei[3], Min Gu[3], Chunchen Zhang[3], Zhengbin Gu[3], Xueyun Wang[2], Christopher Addiego[4], Daining Fang[5,6], Yuefeng Nie[3,*], Jiawang Hong[2,*], Peng Wang[7,*], Xiaoqing Pan[4,8,*]

[1] Department of Applied Physics, The Hong Kong Polytechnic University, Hung Hom, Kowloon 999077, Hong Kong

[2] School of Aerospace Engineering, Beijing Institute of Technology, Beijing 100081, China

[3] National Laboratory of Solid State Microstructures, Jiangsu Key Laboratory of Artificial Functional Materials, College of Engineering and Applied Sciences and Collaborative Innovation Center of Advanced Microstructures, Nanjing University, Nanjing 210093, China

[4] Department of Physics and Astronomy, University of California-Irvine, Irvine, California 92697, USA

[5] Institute of Advanced Structure Technology, Beijing Institute of Technology, Beijing 100081, China

[6] State Key Laboratory for Turbulence and Complex Systems & Center for Applied Physics and Technology, College of Engineering, Peking University, Beijing 100871, China

[7] Department of Physics, University of Warwick, Coventry, CV4 7AL, UK

[8] Department of Materials Science and Engineering, University of California-Irvine, Irvine, California 92697, USA

[#] These authors contributed equally: Songhua Cai, Yingzhuo Lun and Dianxiang Ji.

*Correspondence to: E-mail: songhua.cai@polyu.edu.hk; ynie@nju.edu.cn; hongjw@bit.edu.cn; peng.wang.3@warwick.ac.uk; xiaoqing.pan@uci.edu

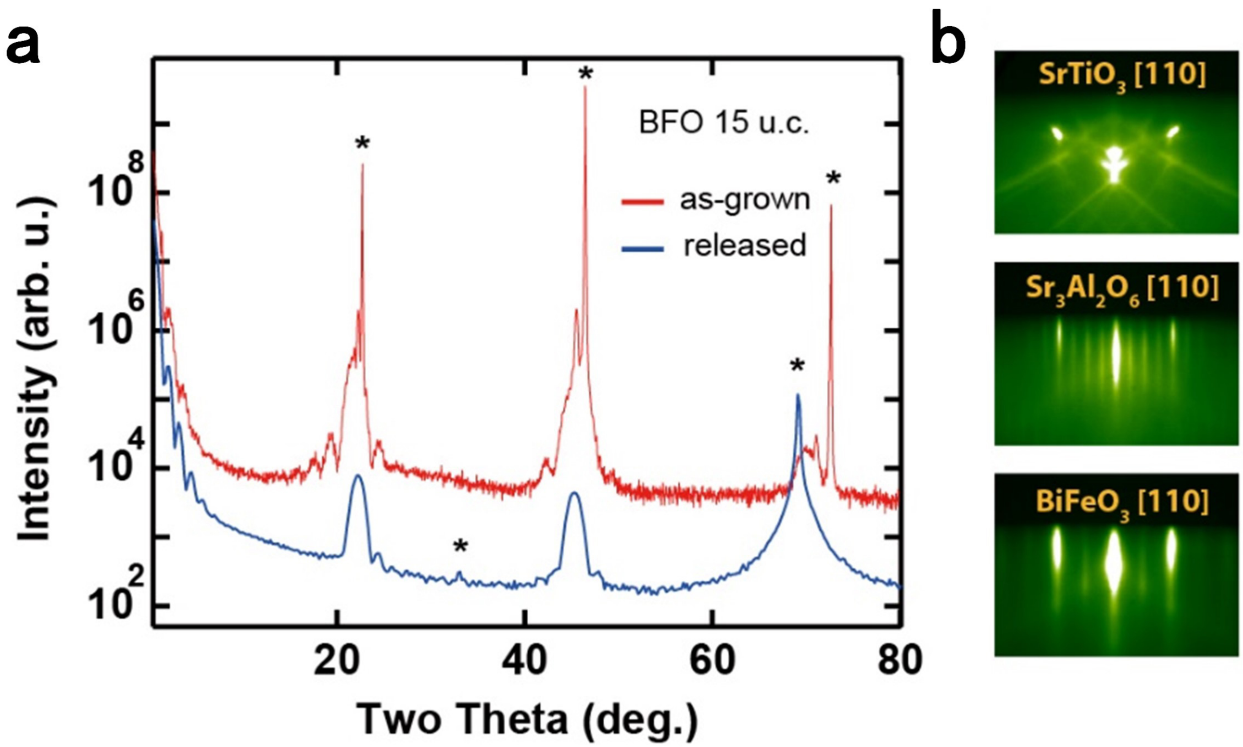

Supplementary Fig. 1 XRD and RHEED results of as-grown 15 u.c. BFO membrane. a, High-resolution XRD 2θ-ω scans of 15 u.c. BFO membrane on STO substrate with SAO buffer layer and the corresponding freestanding BFO film transferred onto silicon substrate. The asterisks denote the background diffraction peak from STO and silicon substrate, respectively. **b,** The RHEED diffraction patterns of STO substrate, SAO buffer layer and BFO membrane, respectively.

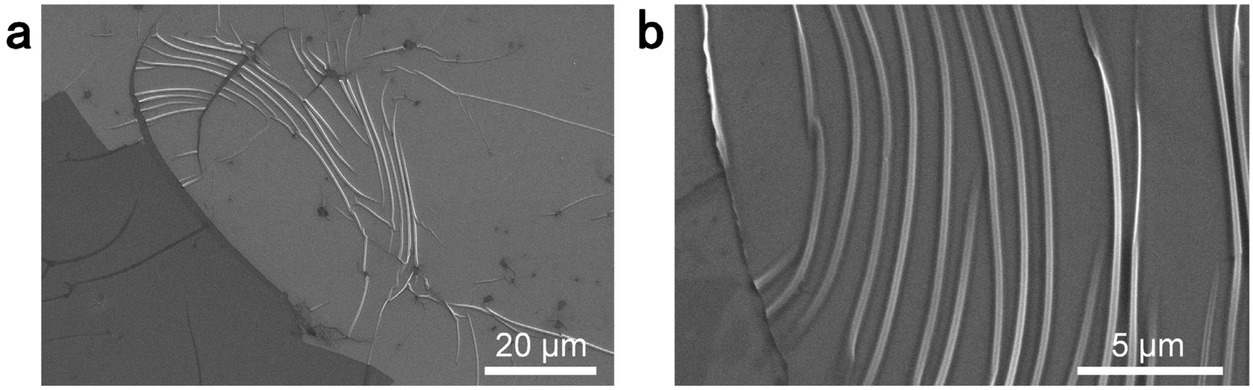

Supplementary Fig. 2 SEM images of typical wrinkled freestanding BFO after transferred onto silicon substrate. a, Wrinkles in 15 u.c. freestanding BFO, with a length of several tens of micrometers. **b,** Wrinkles in 10 u.c. freestanding BFO.

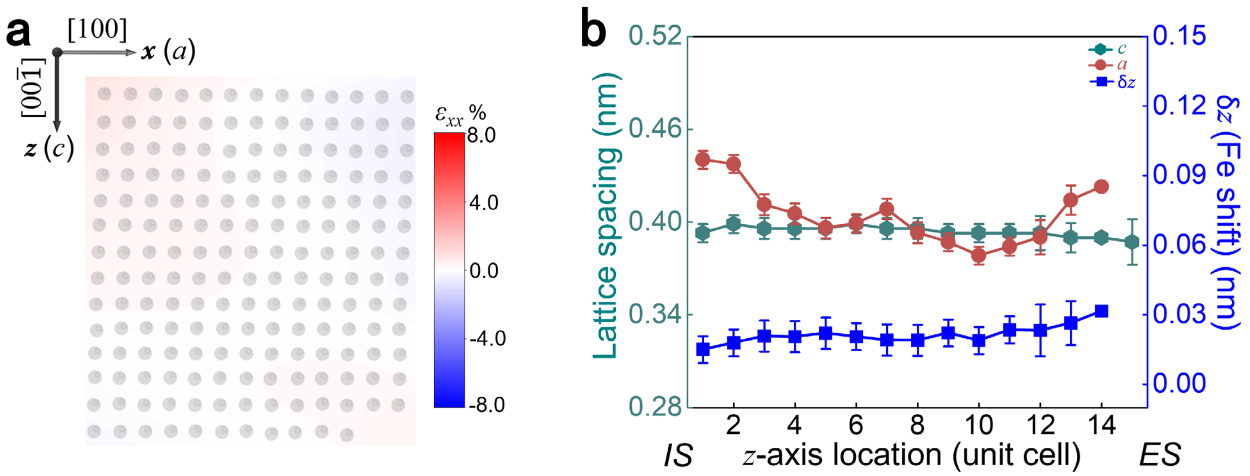

Supplementary Fig. 3 Strain and lattice spacing of unstrained freestanding BFO. a, Distribution of the in-plane strain ε_{xx} in unstrained freestanding BFO mapped from the HAADF image (inset in Fig. 1b). **b,** Line profiles of the variations of lattice spacing *c* and *a*, and the off-centering displacement of Fe cations δ*z* across from the bottom to top surfaces measured from Fig. 1b.

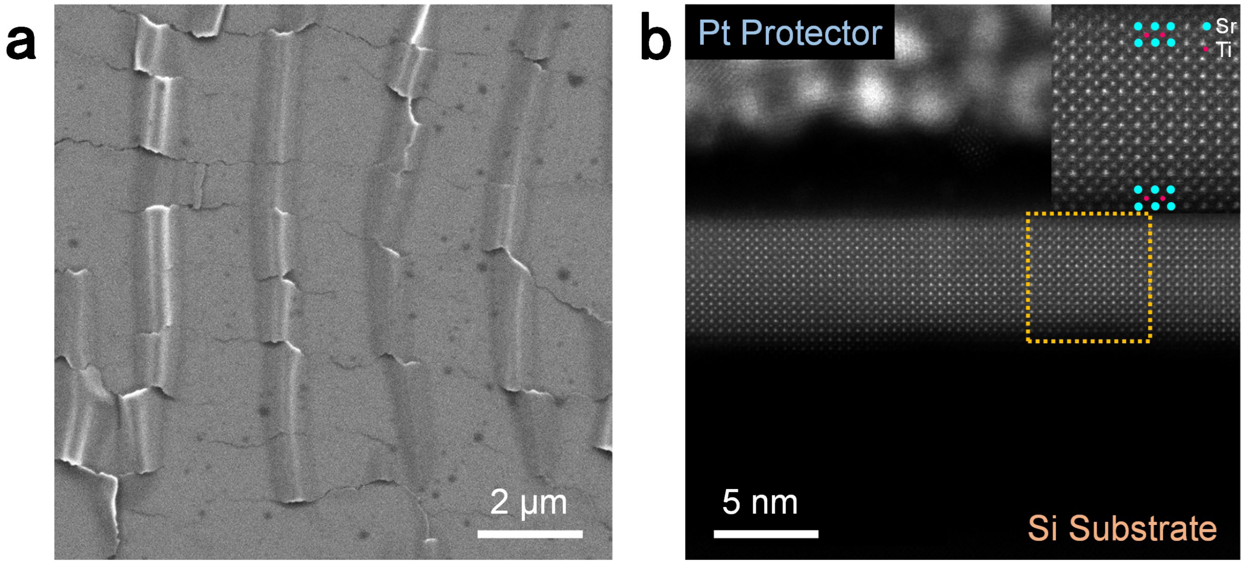

Supplementary Fig. 4 Wrinkle Shape and atomic structure of freestanding STO. a, SEM image of wrinkles in a freestanding STO after transferred onto a silicon substrate. **b,** STEM-HAADF image of unstrained freestanding STO, indicating a typical cubic structure without polarization.

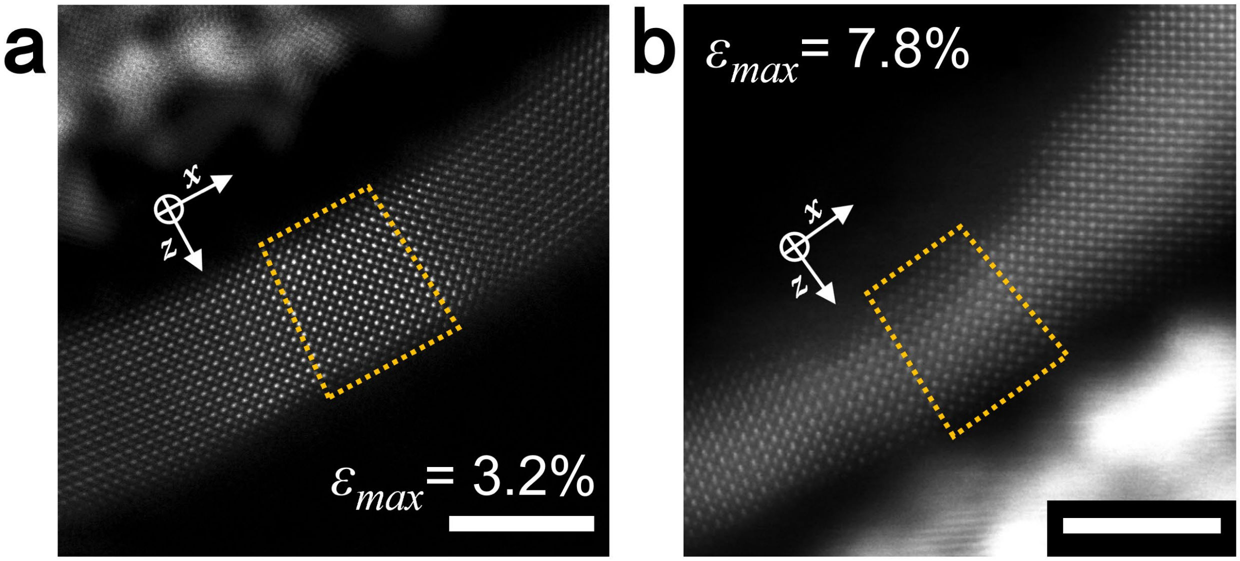

Supplementary Fig. 5 Bent STO membranes under the larger strain gradient. a, b, STEM-HAADF images of freestanding STO taken from the different bent regions with 3.2 % and 7.8 % in-plane strain at external surface, respectively. The strain gradient should be 1.1×10^{7} m^{-1} and 4.3×10^{7} m^{-1} for **a**, **b**, respectively. Under 7.8 % compressive strain, the lattice structure of STO internal surface begins to rupture. Scale bar: 5 nm.

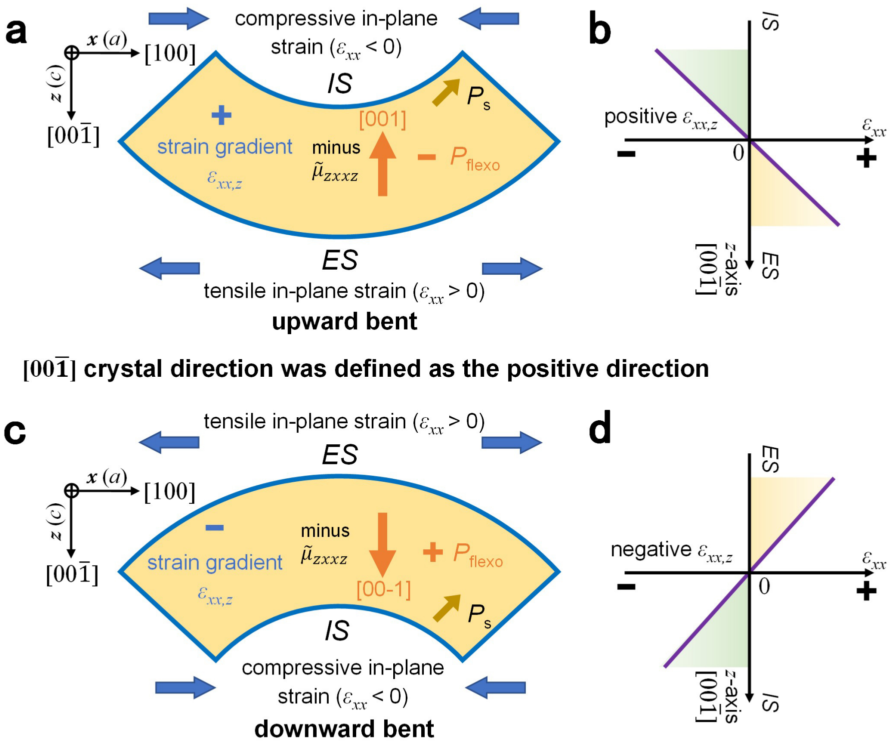

Supplementary Fig. 6 Schematic of defining the [00$\bar{1}$] crystal direction as the z positive direction in bent membranes for the calculations of the strain gradients and polarizations. Here, we define the surface of a bent membrane facing toward the center of bending curvature as internal surface (IS), which suffers compressive in-plane strain with a negative sign, and the opposite surface as external surface (ES), which suffers tensile in-plane strain with a positive sign. Throughout this work, the [00$\bar{1}$] crystal direction of the membranes as shown in Fig. 1 is constantly defined as the positive z direction. Therefore, in the case of bending toward the [001] direction (**a**), the in-plane strain increases from IS to ES along the [00$\bar{1}$] direction (**b**), and therefore, the strain gradient $\varepsilon_{xx,z} > 0$. Whereas, in the case of bending toward the [00$\bar{1}$] direction (**c**), the strain decreases from ES to IS along the [00$\bar{1}$] direction (**d**), and therefore, the strain gradient $\varepsilon_{xx,z} < 0$. Due to the negative sign of the flexoelectric coefficients $\tilde{\mu}_{zxxz}$ for BFO and STO, the flexoelectric polarization calculated always has an opposite sign to the strain gradient. Noted that the positive sign of polarizations indicates that they point toward the [00$\bar{1}$] direction.

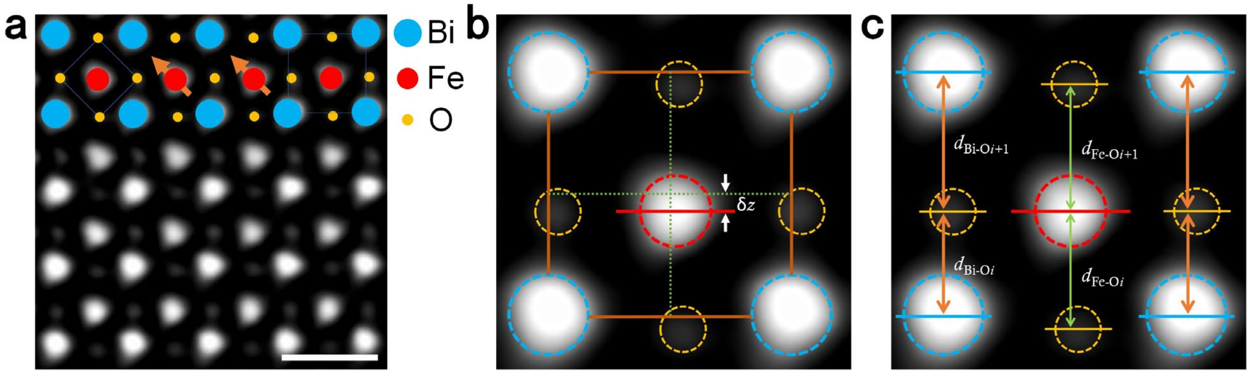

Supplementary Fig. 7 Principle of polarization measurements according to ion displacement. **a,** dCOM image of unstrained freestanding BFO, indicating the real-space position of cations and oxygen ions. A spontaneous polarization with upward out-of-plane component can be revealed. Blue, red and yellow circles represent Bi, Fe and O atomic columns, respectively. Scale bar: 1 nm. **b,** Measurement of relative displacement δz of B site atomic column to center of nearest four A site atomic columns. The out-of-plane polarization P is calculated based on the empirical formula $P = k\delta z$, where k is an empirical constant fitted from macroscopic measurement of corresponding ferroelectric materials[26]. **c,** Precise measurement of out-of-plane polarization based on cation-anion bond length[27], details in **lattice and polarization measurements** in **Methods**. The calculated average out-of-plane spontaneous polarization of freestanding BFO is about 52.7 μC/cm^2.

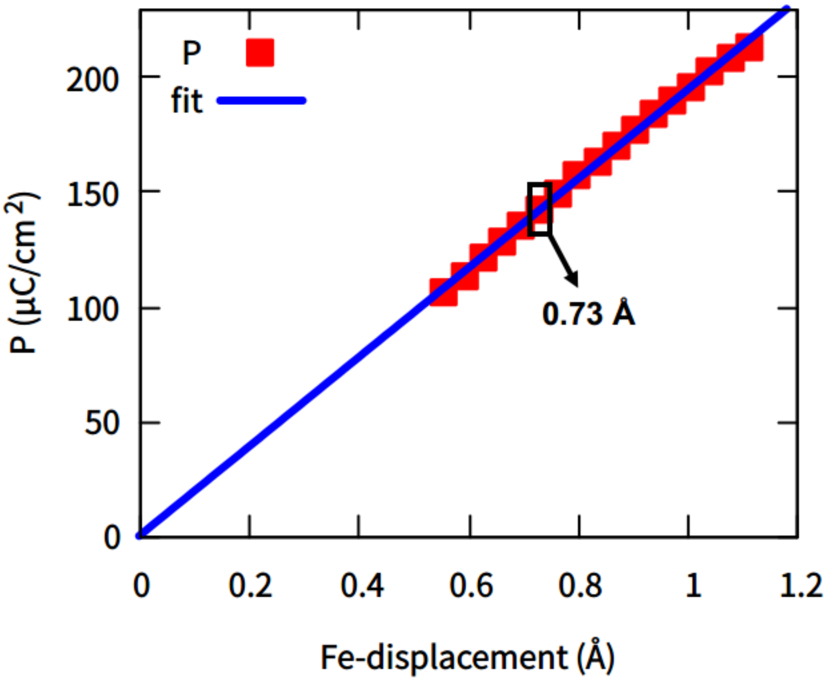

Supplementary Fig. 8 First-principles calculation result of bulk BFO polarization and Fe displacement. The spontaneous polarization of bulk BFO is 144.3 μC/cm^2 along [001] direction (with Fe displacement of 0.73 Å) and it shows a linear change through Fe displacement. The BFO becomes metallic with Fe displacement less than 0.556 Å through the current calculation method, which is consistent with the reported results[40].

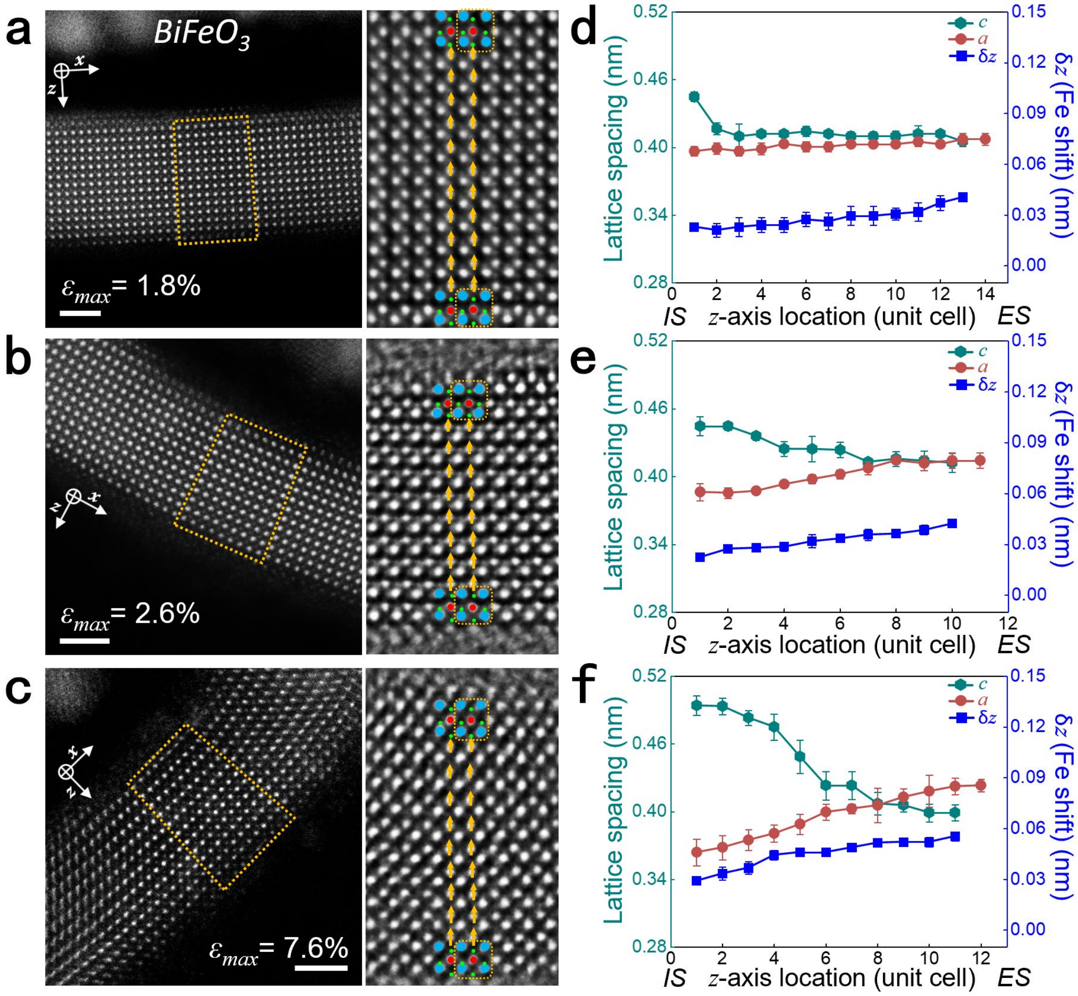

Supplementary Fig. 9 Lattice evolution in bent BFO membranes. a-c, STEM images of bent BFO membranes similar with Fig. 1c-e. **d-f**, Line profiles of the variations of lattice spacing *c* and *a*, and the displacement of Fe cation δ*z* across from the internal to external surfaces in **a-c**, respectively.

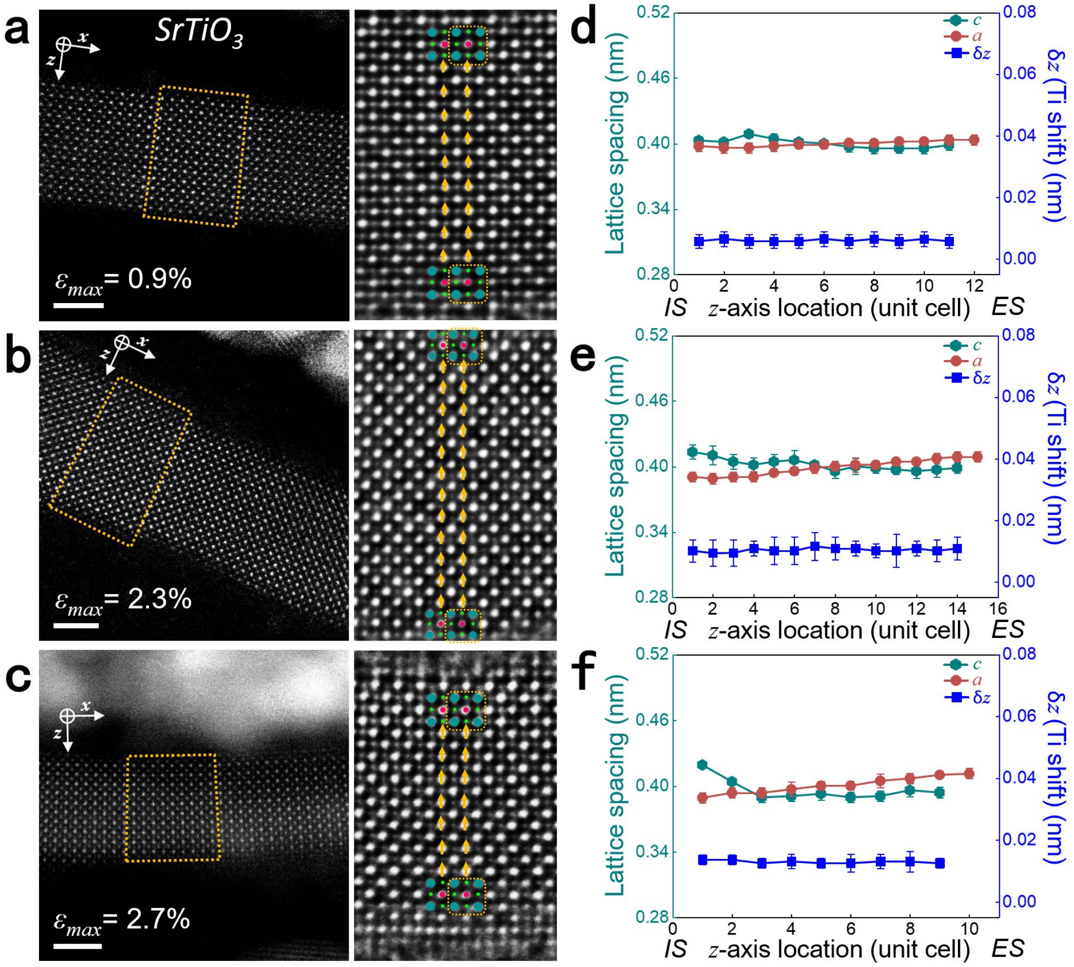

Supplementary Fig. 10 Lattice evolution in bent STO membranes. a-c, STEM images of bent BFO membranes similar with Fig. 2a-c. **d-f**, Line profiles of the variations of lattice spacing *c* and *a*, and the displacement of Ti cation δ*z* across from the internal to external surfaces in **a**-**c**, respectively.

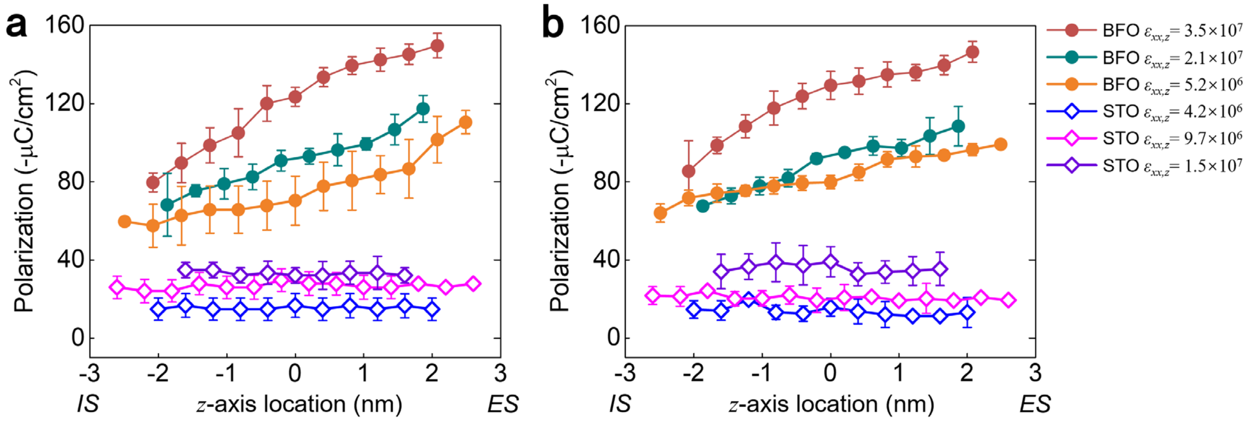

Supplementary Fig. 11 Analysis of polarization distribution along the thickness direction in bent BFO and STO. a, Polarization values calculated from Figs. 1c-e and Figs. 2a-c according to the off-centering displacement of B-site cations. **b**, Polarization values calculated from STEM-iDPC images in Figs. 1c-e and Figs. 2a-c based on the displacement of both cations and oxygen anions.

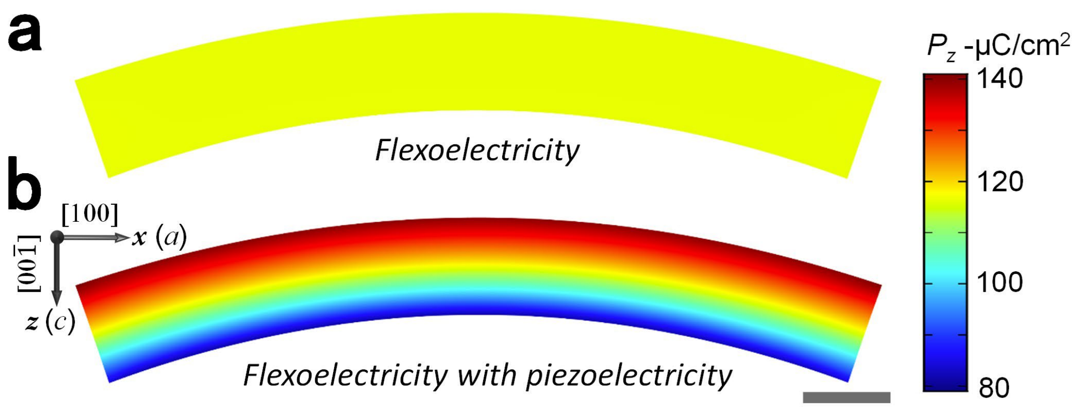

Supplementary Fig. 12 Phase-field simulation of polarization distribution in bent freestanding BFO. a-b, Phase-field simulation results of out-of-plane polarization distribution in bent freestanding BFO with a thickness of 5.0 nm without/with considering piezoelectricity. Scale bar: 5 nm.

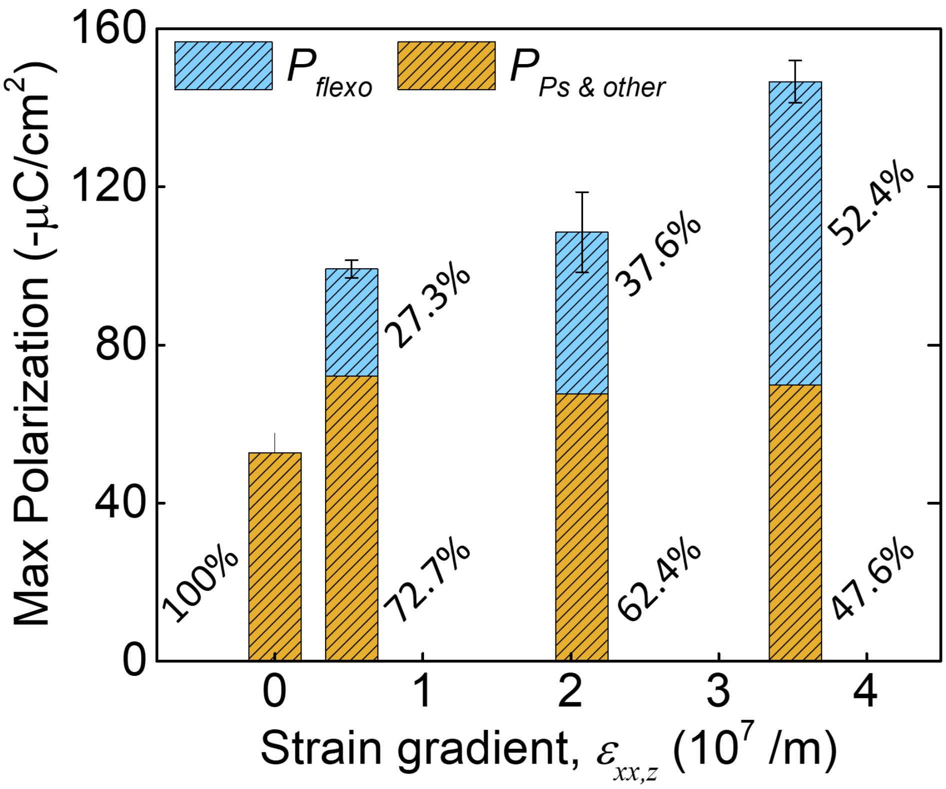

Supplementary Fig. 13 The maximum polarization at the external surface of bent BFO in Fig. 1c-e. P_{flexo} represents the contribution from the flexoelectric polarization, and $P_{Ps\&other}$ represents the contribution from the spontaneous polarization, piezoelectric polarization and other potential effects.

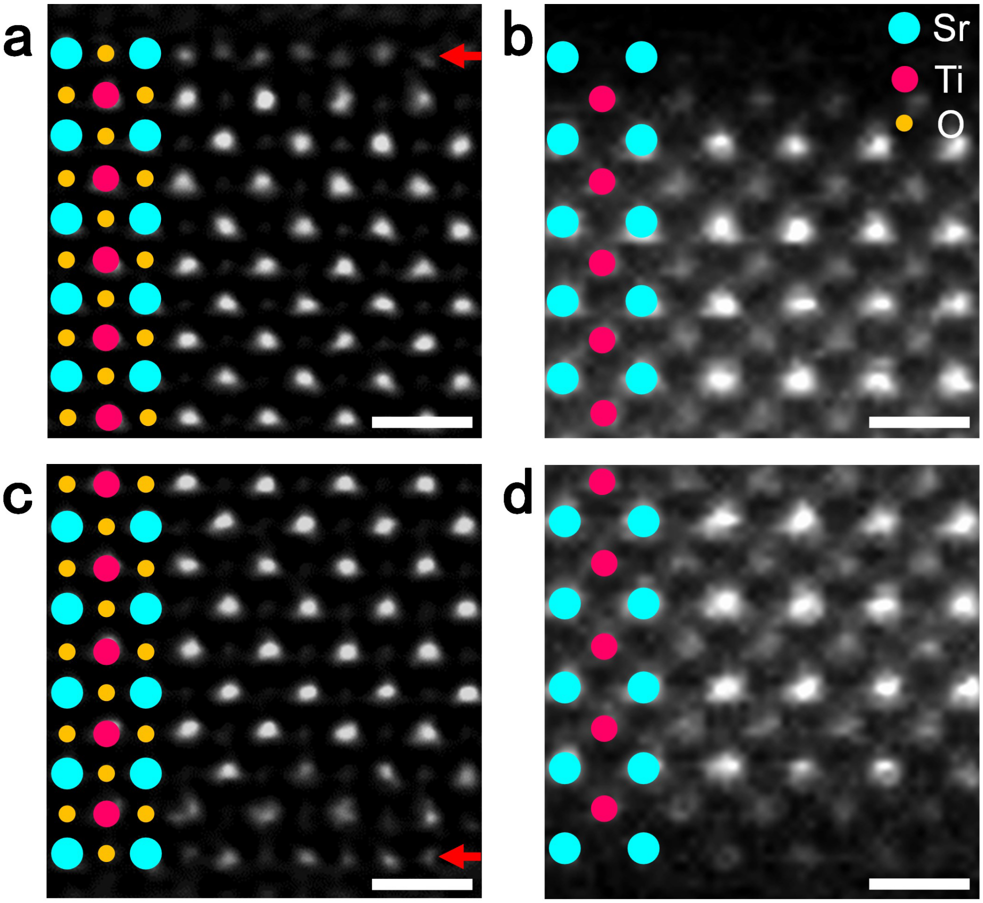

Supplementary Fig. 14 Characterization of termination layers of freestanding STO. a, dCOM image and corresponding HAADF image (**b**) extracted from the same 4D-STEM dataset acquired in top surface region of unstrained freestanding STO. Blue, red and yellow circles represent Sr, Ti and O atomic columns, respectively. Red arrow marked the Sr-O termination layer, which is only visible in dCOM image. **c,** dCOM image and corresponding HAADF image (**d**) extracted from the same 4D-STEM dataset acquired in bottom surface region of unstrained freestanding STO. Both bottom and top surfaces of this freestanding STO are Sr-O terminated. The minus sign of the flexoelectric coefficient of this Sr-O terminated STO measured experimentally is consistent with previous studies on surface control of flexoelectricity[29]. Scale bar: 500 pm.

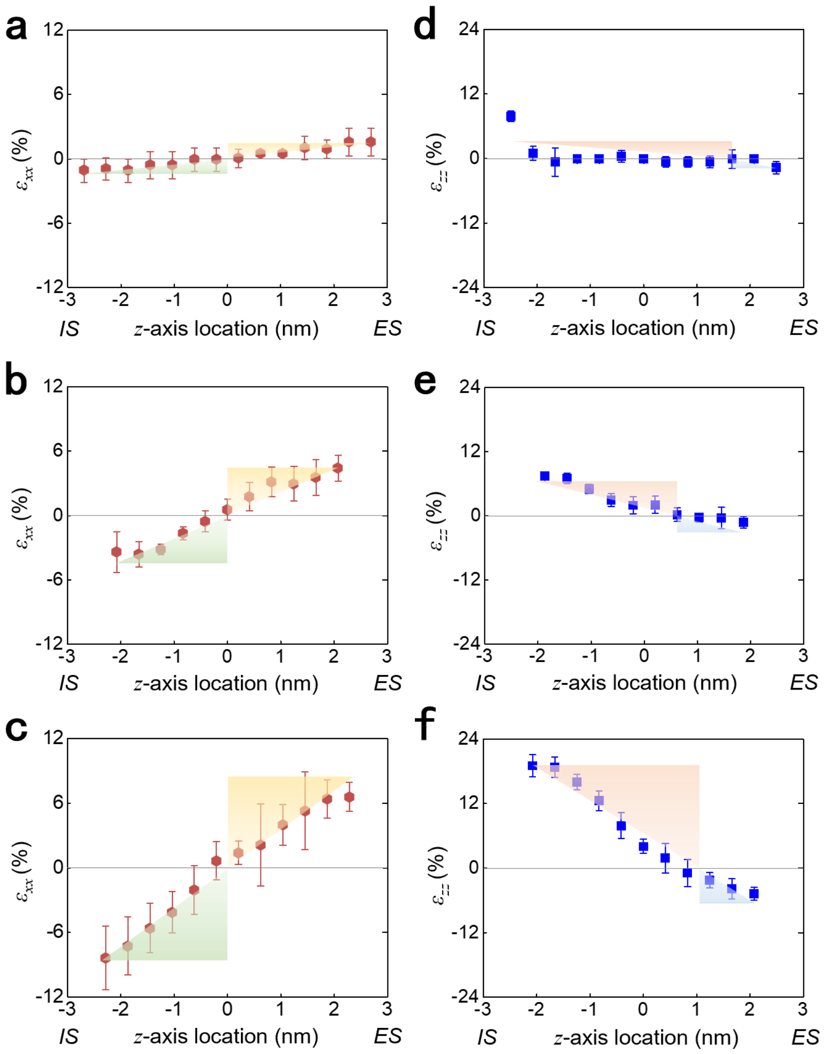

Supplementary Fig. 15 The distribution of in-plane and out-of-plane strain along *z*-axis in bent BFO. a-c, The distribution of in-plane strain ε_{xx} along *z*-axis from internal to external surfaces in bent freestanding BFO in Fig. 1c-e, respectively. **d-f,** The distribution of out-of-plane strain ε_{zz} along *z*-axis from internal to external surfaces in bent freestanding BFO in Fig. 1c-e, respectively.

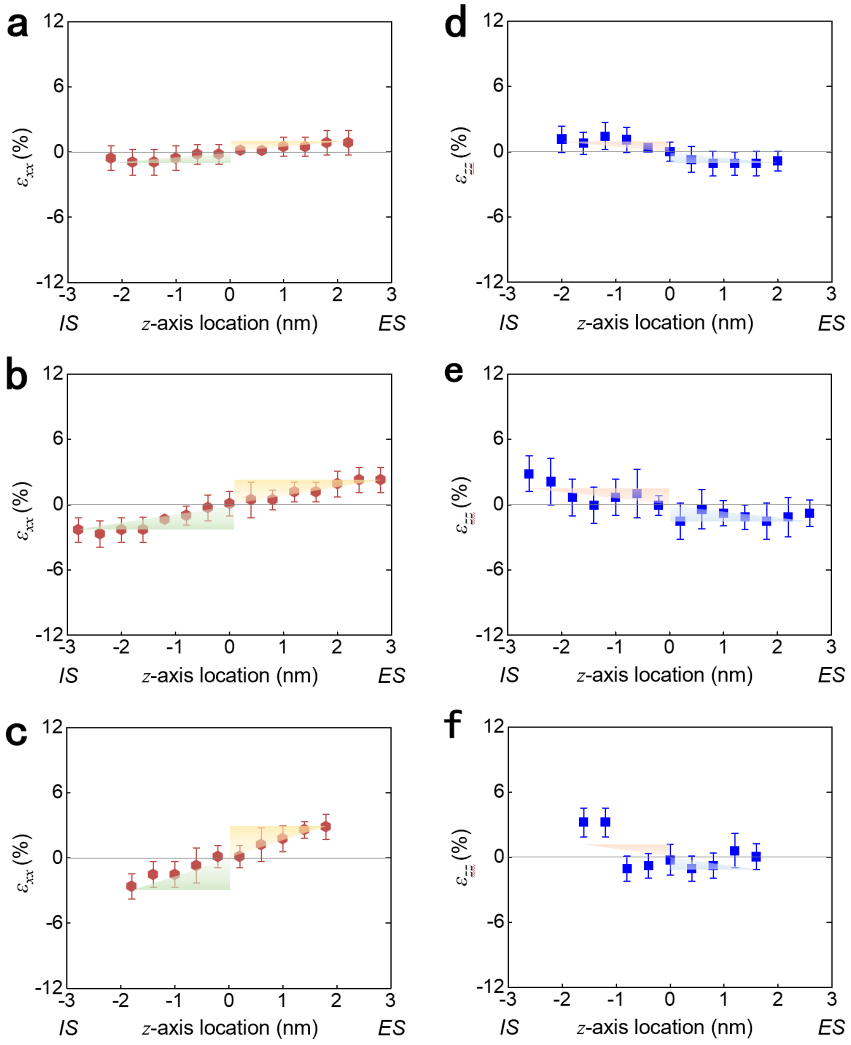

Supplementary Fig. 16 The distribution of in-plane and out-of-plane strain in bent freestanding STO. a-c, The distribution of in-plane strain ε_{xx} along z-axis from internal to external surfaces in bent freestanding STO in Fig. 2a-c, respectively. **d-f,** The distribution of out-of-plane strain ε_{zz} along z-axis from internal to external surfaces in bent freestanding STO in Fig. 2a-c, respectively.

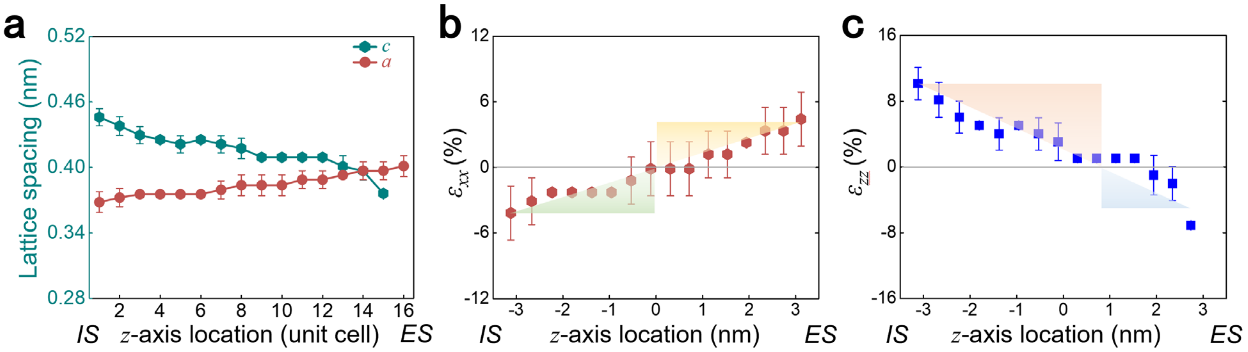

Supplementary Fig. 17 Lattice evolution, in-plane and out-of-plane strain distribution in upward bent freestanding BFO in Fig. 4i. a, Line profiles of the variations of lattice spacing *c* and *a* across from the internal to external surfaces. **b,** The distribution of in-plane strain ε_{xx} along *z*-axis from internal to external surface. The strain gradient is measured as 1.38×10^{7} m^{-1}. **c,** The asymmetric distribution of out-of-plane strain ε_{zz} along *z*-axis from internal to external surface.

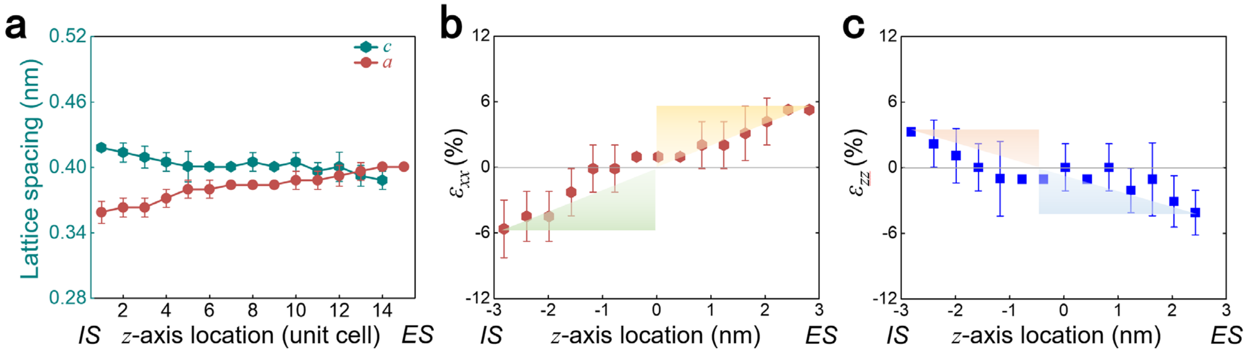

Supplementary Fig. 18 Lattice evolution, in-plane and out-of-plane strain distribution in downward bent freestanding BFO in Fig. 4h. a, Line profiles of the variations of lattice spacing *c* and *a* across from the internal to external surfaces. **b,** The distribution of in-plane strain ε_{xx} along *z*-axis from internal to external surface. The strain gradient is measured as 1.82×10^{7} m^{-1}. **c,** The asymmetric distribution of out-of-plane strain ε_{zz} along *z*-axis from internal to external surface, inversely with Supplementary Fig. 17c.

Supplementary Table 1. The parameters used in the Phase-field simulations.

Parameter	Value	Parameter	Value
α_1	$4.9(T-1103)\times10^5$	G_{11}	$0.6\times G_{110}$
α_{11}	6.5×10^8	e_{31}	17
α_{12}	1.0×10^8	e_{33}	17
c_{11}	3.02×10^{11}	e_{15}	10
c_{12}	1.62×10^{11}	f_{12}	-90
c_{44}	6.8×10^{10}	ε_0	8.85×10^{-12}
G_{110}	0.98×10^{-10}	κ_0	50

(SI units and T in K)
